# Multi-dimensional analysis of oral cavity and oropharyngeal defects following cancer extirpation surgery, a cadaveric study

**DOI:** 10.1186/s40463-018-0276-9

**Published:** 2018-04-24

**Authors:** Sherif Idris, Alex M. Mlynarek, Khalid Ansari, Jeffrey R. Harris, Nabil Rizk, David Cote, Daniel A. O’Connell, Heather Allen, Peter Dziegielewski, Hadi Seikaly

**Affiliations:** 1 Division of Otolaryngology—Head and Neck Surgery, University of Alberta, 1E4 Walter Mackenzie Center, 8440 112 Street, Edmonton, AB T6G 2B7 Canada; 2 Department of Otolaryngology—Head and Neck Surgery, McGill University, Montréal, Quebec, Canada; 3 Department of Otolaryngology—Head and Neck Surgery, University of Florida, Gainesville, Florida, USA

**Keywords:** Oral cavity, Oropharynx, Head and neck, Reconstructive surgery

## Abstract

**Background:**

Defects following resection of tumors in the head and neck region are complex; more detailed and defect-specific reconstruction would likely result in better functional and cosmetic outcomes. The objectives of our study were: 1) to improve the understanding of the two- and three-dimensional nature of oral cavity and oropharyngeal defects following oncological resection and 2) to assess the geometric dimensions and the shapes of fasciocutaneous free flaps and locoregional tissue flaps required for reconstruction of these defects.

**Methods:**

This study was an anatomic cadaveric study which involved creating defects in the oral cavity and oropharynx in two cadaveric specimens. Specifically, partial and total glossectomies, floor of mouth excisions, and base of tongue excisions were carried out. These subsites were subsequently geometrically analyzed and their volumes measured. The two-dimensional (2D) assessment of these three-dimensional (3D) structures included measures of surface area and assessment of tissue contours and shapes.

**Results:**

The resected specimens all demonstrated unique dimensional geometry for the various anatomic sites. Using 2D analysis, hemiglossectomy defects revealed right triangle geometry, whereas total glossectomy geometry was a square. Finally, the base of tongue defects exhibited a trapezoid shape.

**Conclusions:**

Customizing the geometry and dimensions of fasciocutaneous free flaps so that they are specific to the confronted head and neck defects will likely result in better functional and cosmetic outcomes.

## Background

Fasciocutaneous free flaps, such as radial forearm and anterolateral thigh, are commonly used to reconstruct oral and oropharyngeal anatomy following cancer extirpation surgery [1–7]. Most reconstructive surgeons design these free flaps by visually estimating the size of the defect and using basic geometric shapes, such as rectangles, squares or fusiform shapes, to translate the soft tissue flap into the desired form. However, defects following resection of tumors in the head and neck region are considerably more complex. A more detailed and defect-specific reconstruction would likely result in better functional and cosmetic outcomes.

This study endeavors to improve the understanding of the two-dimensional (2D) and three-dimensional (3D) geometric nature of oral cavity and oropharyngeal defects. An enhanced understanding of the dimensional geometry of surgical defects following cancer extirpation surgery will undoubtedly benefit the reconstructive efforts that utilize free and locoregional tissue transfer.

## Methods

**Fig. 1:**
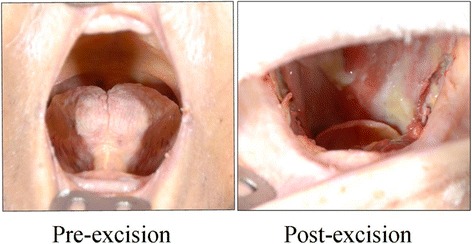
Fresh cadaver pre- and post- total glossectomy: oral tongue, BOT and FOM. *FOM = Floor of mouth; BOT = base of tongue

**Fig. 2:**
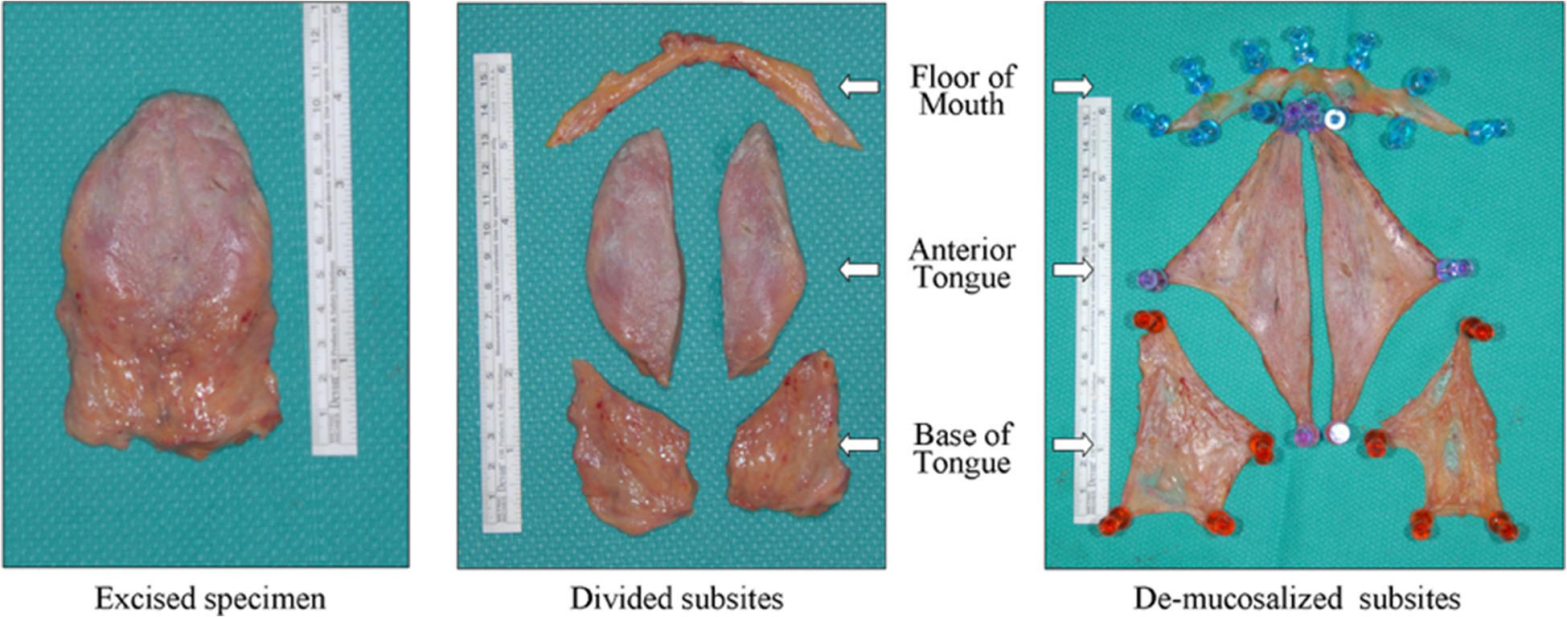
Cadaver total glossectomy specimen was divided into subsites: FOM, anterior tongue and BOT, then de-mucosalized*. *FOM = Floor of mouth; BOT = base of tongue

**Fig. 3:**
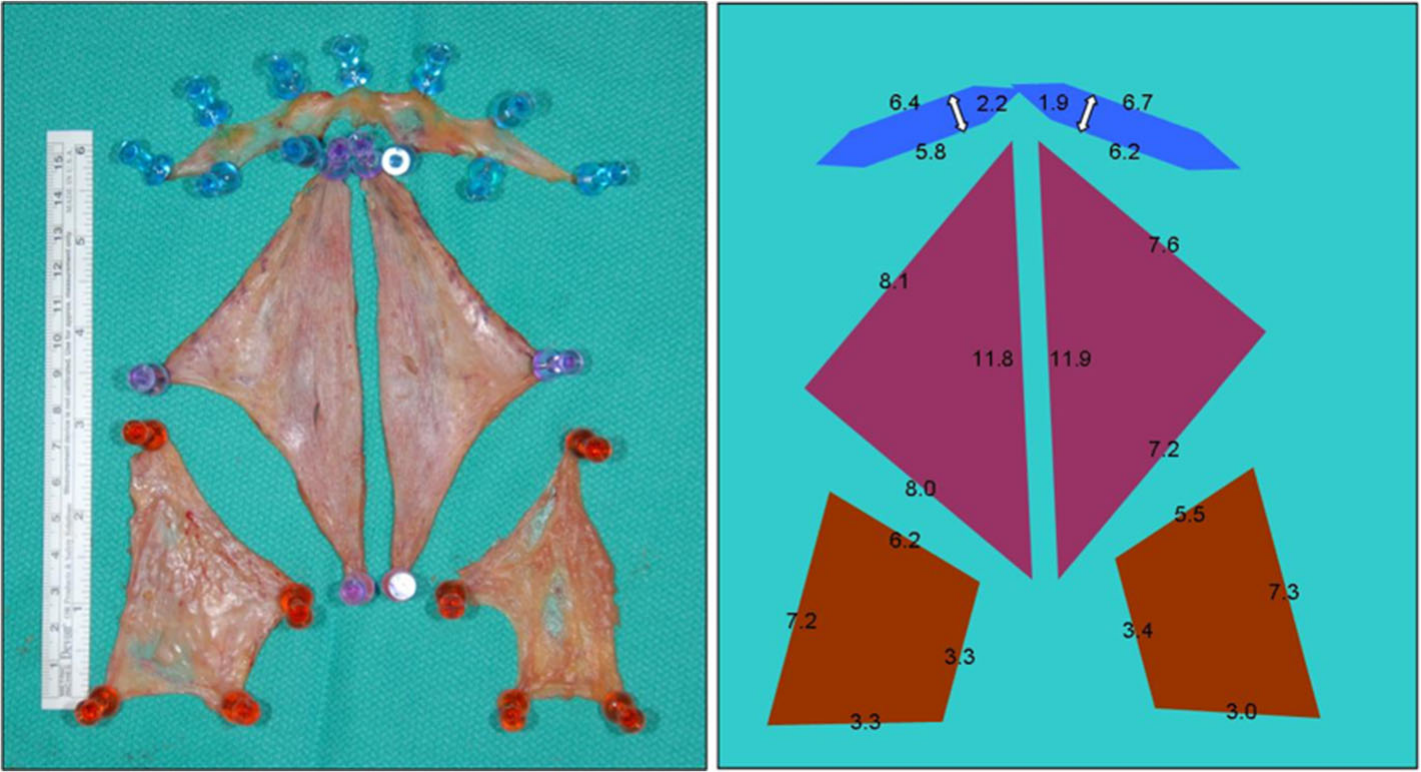
Lengths (cm) of the FOM, anterior tongue, and BOT subsites were measured*. *FOM = Floor of mouth; BOT = base of tongue

**Fig. 4:**
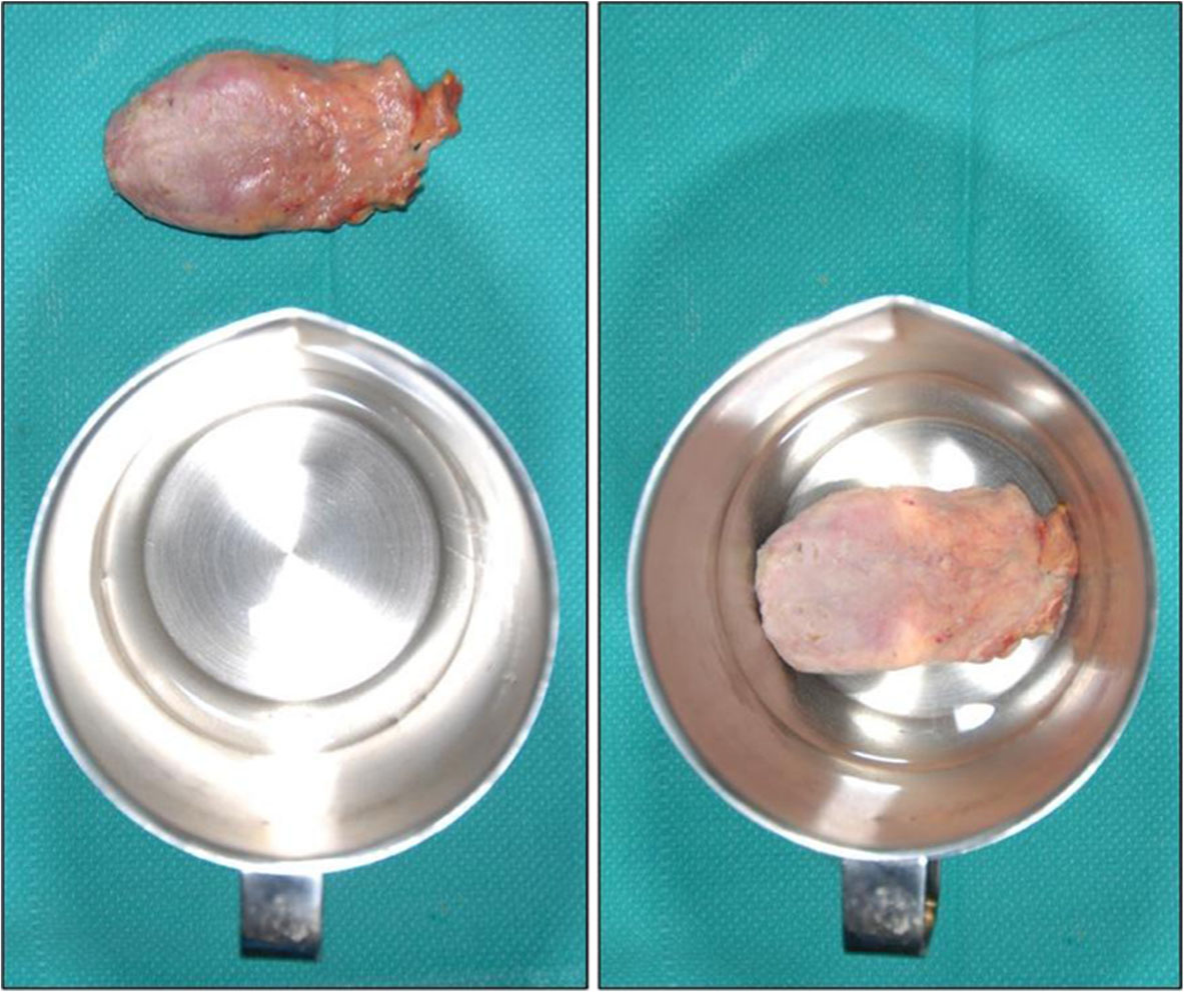
Specimen volumes were measured by amount of water displacement

Institutional Research Ethics Board approval was obtained prior to commencement of the project. This study was conducted at a tertiary-care centre, the University of Alberta Hospital. Two fresh cadavers, one being an 82-year old, 72 kg male and the other being a 90-year old, 60 kg female underwent total glossectomies (Fig. 1). The specimens were then sectioned into specific subsites: floor of mouth, anterior tongue, and base of tongue. Each specimen was subsequently measured for dimensions, volume, and surface area. De-mucosalizing the tissues and spreading them out on a corkboard allowed us to analyze the 2D shape of each specimen (Fig. 2). The component lengths for each resultant geometric shape were measured (Fig. 3). The volume of each subsite was estimated by placing the specimen in a container filled with water and measuring the amount of displaced liquid (Fig. 4).

## Results

The post-excision anterior total glossectomy defect revealed a square shape measuring 8–8 cm, with a volume of about 50 mL. Hemiglossectomy specimens exhibited right triangle geometry, measuring 12–8-8 cm with a volume of 25 mL on average. Hemi-base of tongue specimens revealed a trapezoid shape, measuring an average of 3–3-6-7 cm. There was a high degree of variability in the volume of the base of the tongue, with one cadaver measuring 8 mL and 6 mL for each hemi-base while the other measured 29 mL and 15 mL for each hemi-base. The hemi-floor of mouth specimens were hexagonal in shape, measuring an average of 7 cm in length and 2 cm in width, with a volume of 8.5 mL (Fig. 3). Measurements of specific anatomic structures for each cadaver are highlighted in Tables 1 and 2.

**Table 1:** Measurements from cadaver #1

	Volume (ml)	Height (cm)	Length (cm)	Width (cm)
Tot gloss + BOT + FOM	72	1.75	10.5	6
Tot gloss + BOT	64	1.5	10.5	6
Tot gloss	50	1.5	8	6
Hemi gloss (R)	23	1.5	8	2.5
Hemi gloss (L)	27	1.5	8	3.5
Hemi gloss + BOT (R)	31	1.5	10.5	2.4
Hemi gloss + BOT (L)	33	1.5	10.5	3.2
FOM	8	0.5	7	2
Hemi BOT (R)	8	1.5	2.5	2.8
Hemi BOT (L)	6	1.5	2.5	2.7

*Tot gloss* total glossectomy, *BOT* base of tongue, *FOM* floor of mouth, *Hemi gloss* hemiglossectomy, *L* left, *R* right

**Table 2:** Measurements from cadaver #2

	Volume (ml)	Height (cm)	Length (cm)	Width (cm)
Tot gloss + BOT + FOM	98	1.75	10.5	6
Tot gloss + BOT	89	1.5	10.5	6
Tot gloss	54	1.5	8	6
Hemi gloss (R)	24	1.5	8	2.5
Hemi gloss (L)	30	1.5	8	3.5
Hemi gloss + BOT (R)	43	1.5	10.5	2.4
Hemi gloss + BOT (L)	46	1.5	10.5	3.2
FOM	9	0.5	7	2
Hemi BOT (R)	29	1.5	2.5	2.8
Hemi BOT (L)	15	1.5	2.5	2.7

*Tot gloss* total glossectomy, *BOT* base of tongue, *FOM* floor of mouth, *Hemi gloss* hemiglossectomy, *L* left, *R* right

## Discussion

Reconstruction of head and neck defects following oncologic resection can be challenging. The surgeon must consider both functional and cosmetic outcomes when planning the reconstruction. Swallowing and speech are the chief physiological functions affected by oral and oropharyngeal defects [1, 2, 4, 5, 8, 9]. Numerous reports have been published describing various techniques available to reconstruct these defects [1–3, 10, 11]. Most authors agree that the transfer of a fasciocutaneous free flap is the optimal method for oral cavity and oropharyngeal defect reconstruction. However, considerable debate remains regarding the choice of the donor site and the shape of the flap required for optimal reconstruction. To our knowledge, there are no published anatomical studies that investigate the shape and size of defects and resected tissue following oral and oropharyngeal cancer extirpation surgery.

The results of our study support the practice of creating customized, defect-specific, shaped flaps for oral cavity and oropharyngeal reconstruction after cancer extirpation surgery. For example, the ideal shape to reconstruct a hemiglossectomy defect is a triangle, as we have shown that the 2D defect with this resection is a right triangle. Similarly, a total glossectomy defect would ideally involve the use of a square free flap, and a base of tongue defect would employ a trapezoid-shaped flap for optimal reconstruction. This study is limited by its study size, involving only two cadavers; it is difficult to draw definitive conclusions regarding exact measurements and volumes necessary for these reconstructions. This should be evaluated on a case-by-case basis, depending largely on amount of tissue resected and the size and weight of the patient. Creation of enough bulk and volume for propulsion of the food bolus, while simultaneously protecting the airway, remains of utmost importance when planning the reconstruction of oral and oropharyngeal defects.

## Conclusion

**Fig. 5:**
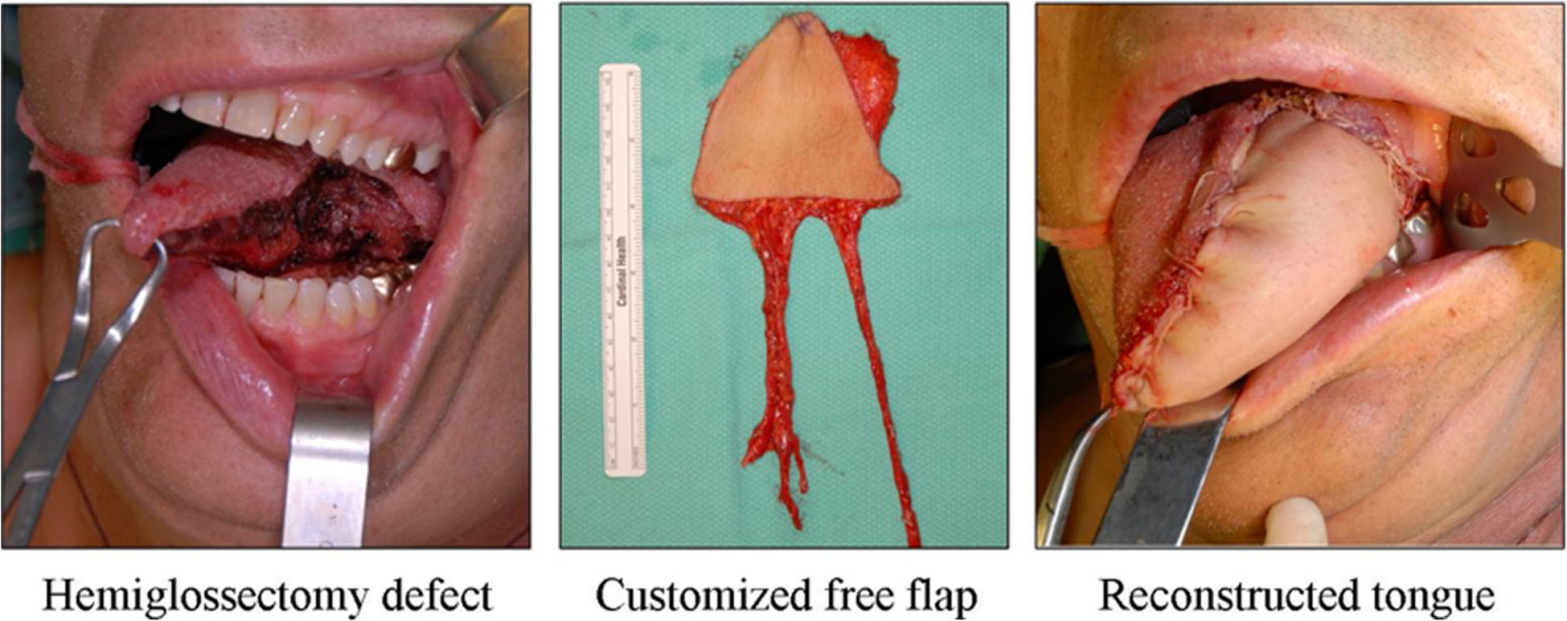
Left anterior hemiglossectomy defect reconstructed with a customized triangular vascularized ulnar free flap

Designing customized fasciocutaneous free flaps that are specifically tailored for the different defects of the oral cavity and oropharynx would likely results in better functional and cosmetic outcomes after reconstruction. For example, an anterior hemiglossectomy defect should be reconstructed with a triangular shaped free flap, such as shown in Fig. 5.

## References

[1] Uwiera T, Seikaly H, Rieger J, Chau J, Harris JR (2004). Functional outcomes after Hemiglossectomy and reconstruction with a Bilobed radial forearm free flap. J Otolaryngol.

[2] Seikaly H, Rieger J, O'Connell D, Ansari K, AlQahtani K, Harris J (2009). Beavertail modification of the radial forearm free flap in base of tongue reconstruction: technique and functional outcomes. Head Neck.

[3] Urken ML, Biller HFA (1994). New Bilobed Design for the Sensate Radial Forearm Flap to preserve tongue mobility following significant Glossectomy. Arch Otolaryngol Head Neck Surg..

[4] Hsiao H-T, Leu Y-S, Liu C-J, Tung K-Y, Lin C-C (2008). Radial forearm versus anterolateral thigh flap reconstruction after Hemiglossectomy: functional assessment of swallowing and speech. J Reconstr Microsurg.

[5] de Vicente JC, de Villalaín L, Torre A, Peña I (2008). Microvascular free tissue transfer for tongue reconstruction after Hemiglossectomy: a functional assessment of radial forearm versus anterolateral thigh flap. J Oral Maxillofac Surg.

[6] Biron VL, O’Connell DA, Barber B (2017). Transoral robotic surgery with radial forearm free flap reconstruction: case control analysis. J Otolaryngol Head Neck Surg.

[7] Orlik JR, Horwich P, Bartlett C, Trites J, Hart R, Taylor S (2014). Long-term functional donor site morbidity of the free radial forearm flap in head and neck cancer survivors. J Otolaryngol Head Neck Surg.

[8] Kimata Y, Sakuraba M, Hishinuma S (2003). Analysis of the relations between the shape of the reconstructed tongue and postoperative functions after subtotal or Total Glossectomy. Laryngoscope.

[9] Dzioba A, Aalto D, Papadopoulos-Nydam G (2017). Functional and quality of life outcomes after partial glossectomy: a multi- institutional longitudinal study of the head and neck research network. J Otolaryngol Head Neck Surg.

[10] Sakuraba M, Asano T, Miyamoto S (2009). A new flap design for tongue reconstruction after total or subtotal glossectomy in thin patients. J Plast Reconstr Aesthet Surg.

[11] Chepeha DB, Teknos TN, Shargorodsky J (2008). Rectangle tongue template for reconstruction of the Hemiglossectomy defect. Arch Otolaryngol Head Neck Surg.

